# Modeling the Significance of Sustainability Orientation on the Sustainability Performance of Micro-Enterprises in an Emerging Economy

**DOI:** 10.3389/fpsyg.2022.881086

**Published:** 2022-05-19

**Authors:** Wan Nurulasiah binti Wan Mustapa, Naeem Hayat, Syed Ali Fazal, Abdullah Al Mamun, Anas A. Salameh, Noor Raihani Zainol

**Affiliations:** ^1^UCSI Graduate Business School, UCSI University, Kuala Lumpur, Malaysia; ^2^Global Entrepreneurship Research and Innovation Centre, Universiti Malaysia Kelantan, Kota Baharu, Malaysia; ^3^Faculty of Business Administration, University of Science and Technology Chittagong, Chittagong, Bangladesh; ^4^UKM-Graduate School of Business, Univeisti Kebangsaan Malaysia, Bangi, Malaysia; ^5^College of Business Administration, Prince Sattam Bin Abdul Aziz University, Al-Kharj, Saudi Arabia; ^6^Faculty of Entrepreneurship and Business, Universiti Malaysia Kelantan, Kota Baharu, Malaysia

**Keywords:** strategic orientation, sustainability orientation, micro-enterprise, performance, sustainability

## Abstract

Sustainable enterprises are essential for developing economies’ progress and prosperity. Micro-enterprises (MEs) play an important role in creating jobs and actively participating in socioeconomic activities. However, little is known about how MEs use internal capabilities to achieve long-term success. This study explores the influence of the strategic orientations (i.e., sales, consumer, competitive, emotional, business, and networking) instigating the sustainability orientation. The sustainability orientation nurtures the sustainable performance of the MEs. The cross-sectional data collected from the 450 MEs with the structured interviews and samples were randomly selected from the list of MEs registered in seven districts of Kelantan, Malaysia. The collected data were analyzed with partial least square structural equation modeling (PLS-SEM). The data analysis discovered that the sale, consumer, competitive, and emotional orientation significantly impact the MEs’ sustainable orientation. The business and networking orientations insignificantly facilitate the sustainable orientation among the samples. The MEs’ sustainable orientation suggestively influences the sustainable performance among the MEs. Furthermore, the study analysis postulated a significant mediating effect of sustainable orientation among the orientations (sale, consumer, competitive, and emotional) on the MEs’ sustainable performance. Our study offers a better understanding of the RBV in the MEs and brings significant empirical evidence to attain sustainable performance with the firm’s level orientation for the MEs. The study brings forward the practical implication for business and policymakers that the MEs require internal and external support to align the business and networking orientation to harness the sustainable performance among the MEs. In the end, the study limitations and future research options are highlighted.

## Introduction

Small and medium enterprises (SMEs) are the levers of economic growth in developed and developing economies (Susilo, 2018). However, the performance of SMEs remains below that of corporate sector firms as the SMEs lack strategic orientation and have a low focus on business performance and sustainability (Hernandez-Perlines et al., 2019). SMEs are unique business units with fewer formalities and often work with limited resources. SMEs around the globe are encountering stringent competition from other SMEs and corporate firms, and SMEs are increasingly becoming less competitive and sustainable due to a lack of resources and orientation (Kaneko and Yimruan, 2017).

The dearth of interest in strategic orientation reflects the lack of concern for the long-term orientation of SMEs and entrepreneurs (Na et al., 2019). The firm-level of strategic orientation manifested in terms of sales, consumer, competitive, emotional, learning, networking, and business orientation (Kim, 2017) can nurture the SMEs’ work and performance. The level of strategic orientation harnesses the sustainability of performing well in today’s and tomorrow’s competitive marketplace (Wang et al., 2015).

Sustainability orientation, on the other hand, is defined as a degree of sustainability that focuses on the social responsibility of the business and performs well for its social and environmental stance (Sung and Park, 2018). Klewitz (2017) postulated that the sustainability orientation indicates the level of anxiety about the protection of the environment along with social responsibility involving elements that assess the fundamental attitudes and behaviors toward the protection of the environment coupled with social concern among European SMEs. Khizar et al. (2022) established that the sustainability orientation helps firms build the necessary paradigm to survive and sustain the business, perform, and make the necessary developmental progression to remain competitive.

### Study Context

Small and medium enterprises are defined in Malaysia as entities with a sales turnover of less than RM 300,000 or employment of less than 5 full-time employees (Bank Negara Malaysia, 2013). Manaf and Ibrahim (2017) documented that the Malaysian government was running different programs that supported small entrepreneurs to become successful and play a significant role in the nation’s development. Malaysia’s governments launched development organizations like Amanah Ikhtiar Malaysia (AIM), Malaysia Fisheries Development Board (LKIM), and The National Entrepreneurs Economic Group Fund (TEKUN), to realize the dream of zero poverty and provide income-generating activities for all.

Earlier studies of sustainability concerned several modes of research focused on specific aspects from the perspective of both developed and developing nations regarding the multiple dimensions of sustainability. Raymond et al. (2013), Adedeji et al. (2019a), and Khizar et al. (2022) suggest that SMEs empower firms’ performance and are necessary for national development. The literature highlights that business firms are taking a keen interest in the sustainability issues in developed economies. However, businesses in developing economies are less inclined toward taking proactive actions toward sustainability (Adomako et al., 2020). As a result, the current study intends to fill in the gaps in the literature, and it integrated strategy and sustainability orientation among Malaysian MEs to investigate sustainable performance from a unique perspective (i.e., firm-level orientation).

Correspondingly, the interplay of strategic orientation and sustainability orientation has been the focus of discussions in forward-thinking economies, with little existing work showcasing the context of emerging countries. Therefore, it is crucial to verify the influence of strategic orientation dimensions that affect MEs’ sustainability orientation and sustainable performances in developing economies, especially in Malaysia. Furthermore, the study is significant for inspiring future studies on the orientation issues concerning the sustainability of MEs in Kelantan, Malaysia, which could improve the socioeconomic position of low-income MEs in Malaysia.

In 2021, SMEs’ contribution to the GDP reached 54.3%, where agriculture, construction, and services are the leading sectors, and SMEs are providing 48.0% of the employment (Department of Statistics Malaysia, 2021). The services and manufacturing sectors have seen a decline due to COVID-19, and efforts are required to maintain the sustainability to handle stressful contingencies like COVID-19. Employment in SMEs declined due to COVID-19 and showed a weakness in the MEs’ sustainability orientation and resilience (Department of Statistics Malaysia, 2021). Thus, SMEs’ performance and sustainability remain critical issues due to their contribution to the national GDP (Chang, 2016). Despite the high level of impact, SME sustainability and performance in developing countries are perceived to be receiving insufficient attention from scholars, which provided the initial impetus for this study.

As a result, it would be intriguing to investigate the sustainability of SMEs with firm internal resources and capabilities. Three questions guide the current research work. 1. Examine the role of internal SME orientation in forming SME’s sustainable orientation. 2. Expose the effect of SME’s sustainable orientation on SME’s sustainable performance. Lastly, evaluating the mediating effect of SMEs’ sustainable orientation between their internal resources capabilities and their sustainable performance is crucial.

Consequently, our research aims to examine the influence of SMEs’ strategic (Sales, Consumer, Competitive, Emotional, Business, and Networking) orientations on the sustainable orientation. The SMEs’ sustainable orientation facilitates their sustainable performance. Furthermore, to evaluate the mediating effect of sustainable orientation between the strategic orientation and the SMEs’ sustainable performance.

## Theoretical Framework and Hypotheses Development

### Theoretical Foundation

Resources-based approaches (RBV) propose that businesses with valued, rare, and incomparable resources perform better than those with unavailable resources (Barney, 1995). The resources help transform the input into outputs and efficiently run the business production process. The firms’ resources might be categorized into property-based and knowledge-based resources (Behnam and Cagliano, 2019). However, Salunke et al. (2019) argued that RBV signifies intangible resources as critical performance drivers. The business’s ability to effectively and efficiently utilize the resources can empower the business to attain competitive advantage (Su et al., 2015). Behnam and Cagliano (2019) postulated that intangible resources (Invisible resources) are usually an assortment of knowledge, skill, network, reputation, and orientation. Hence business viewpoint and work environment are the critical issues determining the operation and formation of an enterprise (Chang, 2016). Therefore, based on the above, we summon the RBV to hypothesize the effect of various strategic orientation dimensions (rear and valuable intangible resources) in developing superior enterprise sustainability (sustainable competitive advantage) through the mediation of sustainability orientation (as a unique competence).

### Strategic Orientations and Sustainability Orientation

#### Sales Orientation and Sustainability Orientation

The first dimension of the strategic orientation paradigm considered in this study was sales orientation which related to the entrepreneur’s engagement to engage in business activities, emphasizing acquiring the income from the sale of products and services (Panagopoulos et al., 2017). Sales orientation captures the entrepreneurs’ ability to develop long-term relationships with their consumers to increase the enterprise’s income (Taneja and Toombs, 2014). The sales orientation creates a practical solution for developing long-term connections with consumers and achieving sales through positive ordinary sales methods (Saengchai and Jermsittiparsert, 2019). Stimulatingly, Panagopoulos et al. (2017) revealed that the firms’ engagement with sales tactics supports the firm in evaluating consumer demands; recommending products that fulfill consumers’ requirements; changing sales appearance to balance consumer interest; and preventing unreliable or manipulative approaches, particularly by avoiding high-pressure selling techniques. The influence on sales orientation could facilitate higher sales, leading to sustainable performance for the entrepreneurial firms (Moura-Leite et al., 2014). Hence, this study likes to offer the following hypothesis:

*Hypothesis 1: Sales orientation has a significant positive effect on sustainability orientation.*

#### Consumer Orientation and Sustainability Orientation

Consumer orientation emphasizes the entrepreneurs’ efforts to identify the consumer’s needs and wants by assisting the consumer in finding the best-suited alternative to select and offer the appropriate solution to the consumer (Khan and Khan, 2019). Ajayi (2016) specified that SMEs, which boost consumer-orientated behavior in their industry, can transform the desire and preferences of the existing and future consumers, gaining an advantage in the future competition using the assistance of an advanced consumer orientation method. Wang et al. (2016) differ and stated that consumer orientation is simply meeting the consumer needs as the primary function of the business. Thus, customer orientation involves knowing how consumers generate and deliver value-added products and services. Firms’ level of customer orientation instigates the firm-level resource that nurtures the competency to attain a sustainable mind (Behnam and Cagliano, 2019). Furthermore, Kim (2017) specified that consumer orientation leads to higher business income, thereby having a positive effect on the performance of the business. Henceforth, the following hypothesis is proposed:

*Hypothesis 2: Consumer orientation has a significant positive effect on sustainability orientation.*

#### Competitive Orientation and Sustainability Orientation

Competitive orientation refers to the firm’s engagement with the activities to change the knowledge-based business resources and operational practices (Salunke et al., 2019). Adedeji et al. (2019b) specified that the innovative resource-combinations sustenance the firm to pursue its primary resource generating and innovatively utilizing the resources to resolve the problems and adopt changes or expected changes to compete well in the market. Consequently, competitive orientation refers to the business skill and capabilities required to achieve a superior marketplace position (Salunke et al., 2019). Combining knowledge resources stimulates competitive orientation, nurtures adaptability and flexibility, and offers the best consumer products and services (Behnam and Cagliano, 2019). The competitive mindset empowers firms to build tailor-made solutions to remain competitive and achieve sustainability. Businesses generate complicated solutions for their consumers that regularly require integrating diverse knowledge-based resources (Ajayi, 2016). Therefore, we propose the following hypothesis:

*Hypothesis 3: Competitive orientation has a significant positive effect on sustainability orientation.*

#### Emotional Orientation and Sustainability Orientation

Emotional orientation reflects another dimension of strategic orientation substantial for the firm (Berrone et al., 2012). As humans run firms, it is logical to expect that emotions play a significant part in business management and affect the decision-making of people involved (Chang, 2016). In addition, emotion plays a vital role in creating new business and is vital for the firms’ sustainability (Jones et al., 2017). The attachment called emotions typically leads to increased creativity and the right imagination power to handle risky situations (Berrone et al., 2012). The emotions authorize the firms to augment creative business behaviors, sustain the business, and recognize opportunities in risky environments (Kim, 2017). Recently, Hernandez-Perlines et al. (2019) revealed that emotional orientation significantly influences business performance. Henceforward, the following hypothesis was presented:

*Hypothesis 4: Emotional orientation has a significant positive effect on sustainability orientation.*

#### Business Orientation and Sustainability Orientation

Business orientation deals with sustainability issues related to the two aspects; first, the ability for the business entities to make profits, ensuring survival, and the capability of business entities to deliver products and/or services based on procedure or technology that facilitates the environment and/or society (Fernando et al., 2019). The business-oriented firms are keenly engrossed in obtaining information to improve skills to predict current business movements and expose the consumer’s needs based on the latest technology without harming the environment (Choongo et al., 2016). Adedeji et al. (2017) claimed that the firm’s capacity to decode consumer needs reflects the business orientation and helps the firm sustain performance. However, a business needs to be open to social and environmental needs while achieving the long-term goals of the firm. Therefore, the following hypothesis is offered:

*Hypothesis 5: Business orientation has a significant positive effect on sustainability orientation.*

#### Networking Orientation and Sustainability Orientation

Small and medium enterprises must expand their relationship with the workers, consumers, and competitors, thus improving their business by stimulating creativity in bonding with their stakeholders (Adiguzel, 2019). Su et al. (2015) revealed that the networking orientation, as a type of managerial networking ability, depicts a positive moderating effect on the linkage between entrepreneurial orientation and firm performance. Strengthening the network ties improves entrepreneurship in business, thereby increasing the business’s overall performance (Martins, 2016). Ajayi (2016) established a significant association between networking and firm performance. Setya et al. (2017) described that the firms’ level of networking empowers the firms to achieve sustainability; the study shows that expanding the strength, opportunities, and approaches in networking affects business sustainability. Jones et al. (2017) endorsed that SMEs develop extensive networking; networking helps the business to increase business performance through absorbing innovation and enhancing business sustainability. We like to offer the following hypothesis:

*Hypothesis 6: Networking orientation has a significant positive effect on sustainability orientation.*

#### Sustainability Orientation and Micro-Enterprise Sustainability

Sustainability orientation is the latest causal link to marketing a business that reduces the environmental devastation caused by the business production processes (Adedeji et al., 2019b). The sustainability-oriented individuals produce products without undermining the environment or society at large; the firm can take the opportunity of continuity and have an advantage over traditional competitors (Choongo et al., 2016). Theoretically, sustainability orientation is enacted as a business strategy that signifies the enterprise viewpoint of organizing and presents an enterprise in a sustainable shape (Sung and Park, 2018). However, sustainability orientation costs include protecting the ecosystem, supporting safety and health, and addressing end of product life recycling concerns (Chang, 2016). Sustainability orientation assists the business to enrich the exhibition of the business’s social image, improves the business’s performance in terms of brand, and declines the reputational risk (Jones et al., 2017). Choongo et al. (2016) mentioned that the sustainability orientation positively affects business sustainability concerning the workers, diversity, society, and environment. Adedeji et al. (2019b) mentioned that sustainability influences business aspects by confirming strengthened market value, generating investment demand, and making the business more attractive. Thus, in the light of the preceding, the following hypothesis is drawn:

*Hypothesis 7: Sustainability orientation has a significant positive effect on micro-enterprise sustainability.*

### Mediating Effect of Sustainability Orientation

Sales orientation focuses on acquiring, building, and sustaining long-term relationships to achieve higher sales performance (Taneja and Toombs, 2014). Sales orientation is the actual process of interaction between the buyers and enterprise to maintain the SMEs’ performance (Panagopoulos et al., 2017). Sales orientation empowers the firm to nurture a long-term relationship between enterprise and consumer to have profitable sales via productive collaborative sales activities (Na et al., 2019). Panagopoulos et al. (2017) declared that the sales orientation helps the enterprise cope with and resolve conflicts. However, for this study, we expect that the sustainability orientation will mediate the correlation of sales orientation with SME sustainability. Therefore, we forwarded the following hypothesis:

*Hypothesis HM1: Sustainability orientation mediates the relationship between sales orientation and micro-enterprise sustainability.*

Kim (2017) documented the positive impacts of consumer orientation on enterprise performance. Through advanced methods, enterprises that support consumer-oriented behavior can employ the changing requirements and preferences of their existing and potential customers ahead of the competition (Saengchai and Jermsittiparsert, 2019). The enterprise with workers focused on consumers continually engages in activities such as collecting, analyzing, and processing information about the consumer and serving them by being proactively innovative (Kim, 2017). The firm-level consumer orientation helps the firm offer sustainable products and services to attain firm sustainability (Ngo and O’Cass, 2012). Khan and Khan (2019) revealed that the firm with the consumer orientation empowers the firm to be productive and positively instigates the business to achieve sustainability. The higher consumer orientation facilitates the consumers’ needs and wants well and offers the right solution to satisfy the customer and gain the firms’ superior performance (Na et al., 2019). Thus, the following hypothesis is drawn:

*Hypothesis HM2: Sustainability orientation mediates the relationship between consumer orientation and micro-enterprise sustainability.*

Firms acquire competitiveness by having the cost advantages by achieving lower operating costs than the competitors while offering comparable products (Klaprabchone et al., 2019). The competitiveness builds the relevant skill to integrate and create value-added products that sustain the resources through supporting market situations relative to rivals (Klewitz, 2017). Adedeji et al. (2019b) specified that the competition positively affects consumer cooperation, leading to enhanced corporate performance. The worldwide assessment helps to understand, acting as a critical factor of market competitiveness, thus being crucial for the SMEs to sustain their growth and performance (Kim, 2017). Businesses must be more market-responsive to aggressive competitor actions, necessitating competitive tactics to please customers in a long-term manner (Thaiprayoon et al., 2019). We like to forward the following mediating hypothesis:

*Hypothesis HM3: Sustainability orientation mediates the relationship between competitive orientation and micro-enterprise sustainability.*

Emotional orientation consists of three central dimensions of adaptive capabilities: appraisal and expression of emotion, regulation of emotion, and utilization of emotions in solving problems (Berrone et al., 2012). Furthermore, emotional orientation also plays a crucial task in forming new or sustaining existing businesses (Kim, 2017). Hernandez-Perlines et al. (2019) recently highlighted that there are favorable links between emotions and business-level orientations, which can lead to improved business success. As a result, emotional management contributes to the business’s long-term viability. Hence, we like to propose the following hypothesis:

*Hypothesis HM4: Sustainability orientation mediates the relationship between emotional orientations on micro-enterprise sustainability.*

Business-oriented firms generating superior consumer value than competitors are always the prime goal while formulating and applying strategies (Na et al., 2019). Fernando et al. (2019) poised that the business orientation helps firms satisfy consumer needs. Moreover, Moura-Leite et al. (2014) stated that business orientation reflects firms’ responsiveness to meet consumer needs and wants, enabling firms to perform better than competitors. Na et al. (2019) extended the previous views proposing that business orientation is perceived as discovering and understanding the consumer need and wants not only for existing consumers but also for potential customers and working to perform better for the sustainability of the business. Business orientation allows the firms to focus on resources that empower firms in resolving issues and finding new opportunities for sustained business performance (Adiguzel, 2019). Therefore, we propose the following:

*Hypothesis HM5: Sustainability orientation mediates the relationship between business orientation and micro-enterprise sustainability.*

Su et al. (2015) revealed that business networking positively moderates the association between sustainability orientation and firm performance. Martins (2016) analyzed and concluded that networking positively moderates the correlation between entrepreneurial orientation and firm performance. However, Setya et al. (2017) exposed that forming firm-level networking orientation strategies helps nurture business-level sustainability. Similarly, Jones et al. (2017) documented that networking is essential for forming and executing business strategies, leading to business sustainability. Klewitz (2017) restated that a significant relationship exists between networking and business sustainability. However, the sustainability orientation requires vital networking among the stakeholders and can improve the firm’s sustainability performance (Jones et al., 2017). The firm-level sustainability orientation may mediate the relationship between its networking and performance. Henceforth, we like to propose the following:

*Hypothesis HM6: Sustainability orientation mediates the relationship between networking orientation and micro-enterprise sustainability.*

## Methodology

**FIGURE 1:**
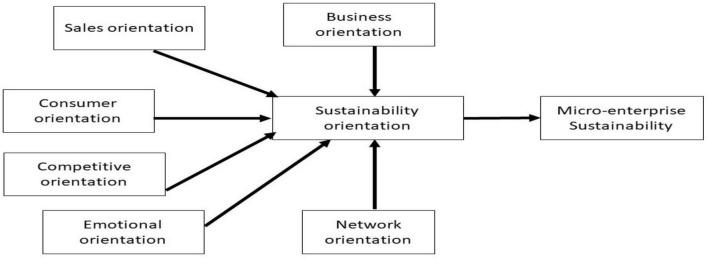
Research framework.

This study used a cross-sectional design to arrange a face-to-face structured interview to collect quantitative data to evaluate the effects of sales orientation, consumer orientation, competitive orientation, emotional orientation, business orientation, and networking orientation on sustainability orientation SMEs’ sustainability in Kelantan Malaysia. Associations hypothesised and tested are presented in Figure 1. The population for the study was 88,435 low-income SMEs identified as participants of the development program initiated by TEKUN, AIM, and LKIM in Kelantan, Malaysia. We obtained three randomly selected participants from three development organizations, i.e., a list of 500 low-income SMEs from AIM Kelantan, 350 low-income SMEs from TEKUN, and 156 low-income SMEs from LKIM Kelantan. A total of 1,006 active participants were located in seven districts, namely Tumpat, Bachok, Pasir Puteh, Pasir Mas, Tanah Merah, Gua Musang, and Jeli. At the start of data collection, the research team called all 1,006 respondents to explain the purpose of the survey and to secure interview appointments. However, 450 people were questioned to complete the survey questionnaire, and the final analysis was done.

### Sample Size

G-Power version 3.1 was used to calculate the sample size for this study. To test the research model with seven predictors, a sample size of 153 was necessary, based on a power of 0.95 and an effect size of 0.15 (Faul et al., 2007). However, to address any potential limitations resulting from the limited sample size, we gathered quantitative data from 450 SMEs in nine districts across Kelantan, Malaysia.

### Common Method Variance

To address the issue of common method-related bias, we cautiously constructed the items. Also, we informed the respondents that “*they were evaluated anonymously and there were no right or wrong answers*” (Podsakoff et al., 2003). Furthermore, as Podsakoff et al. (2003) suggested, this study adopted a five-point Likert scale for all independent variables and a seven-point Likert scale for the dependent variable. In addition, for statistical remedy, this study adopted Harman’s (1976) one-factor test. One fixed factor extracted from all principal constructs is expected to explain less than 50% of the variance. The results show that one component explained 27.50% of the variance, which is less than the maximum threshold of 50%. Furthermore, correlation among constructs that exceed 0.9 is considered an indicator of common method bias (Kock, 2015). For the current study, the highest correlation between the constructs was 0.804 (between consumer orientation and competitive orientation), thus indicating no serious issue of common method bias in the collected data.

### Research Instrument

The questionnaires were designed using simple terms and particular question items adapted from earlier studies with minor alterations to assess latent constructs appropriately. The items employed to measure sales orientation were adopted from Periatt (2004). The consumer orientation was estimated with the question items retrieved from Ramani and Kumar (2008), whereas question items evaluating the competitive orientation were adopted from Gonzalez-Benito et al. (2009). Schutte et al. (1998) assumed the items estimating the emotional orientation. In addition, the question items gauging the business orientation were espoused by Gonzalez-Benito et al. (2009). However, the networking orientation was evaluated with the questions items taken from Witt (2007). The sustainability orientation was assessed with the question items adopted from Roxas and Coetzer (2012). Finally, SME sustainability was appreciated with the questions items assumed by Raymond et al. (2013). All items and sources presented in Appendix 1. A seven-point Likert scale (1 to 7, from “strongly disagree” to “strongly agree”) was used for SME sustainability. In contrast, a five-point Likert scale (1 to 5, from “strongly disagree” to “strongly agree”) was employed for all the independent variables.

### Multivariate Normality

Structural equation modeling-partial least squares (SEM-PLS) is not associated with multivariate normality in the data as it is a non-parametric analysis instrument (Hair et al., 2019). Following the recommendation of Peng and Lai (2012), multivariate data normality was tested using an online tool of web power^1^ to confirm data normality. The test outcomes approved that the data set is not as normal as Mardia’s multivariate coefficient *p*-values of less than 0.05 (Cain et al., 2017).

### Data Analysis Method

Partial least squares structural equation modeling (PLS-SEM) was used with the Smart-PLS software 3.1 for data analysis. PLS-SEM is a multivariate analysis instrument to gauge the path models with composites’ latent constructs (Hair et al., 2019). PLS-SEM empowers the researcher to tackle the non-normal and small data sets. Furthermore, PLS-SEM has a casual-predictive nature with an undisturbed supposition of goodness-of-fit estimation than the covariance-based SEM (Chin, 2010). Two-step techniques analyzed data with PLS-SEM, and the first measurement was performed to test the model’s reliability and validity at the constructs’ level (Hair et al., 2019). The second stage was executed to estimate the structural model and investigate study hypotheses with significance levels (Chin, 2010). Model estimation was performed with *r*^2^, *Q*^2^, and the effect size *f*^2^ describing the path effect from the exogenous construct for the endogenous construct (Hair et al., 2019).

### Demographic Characteristics

A total of 450 valid responses were collected for this study, representing the SMEs working in Kelantan, Malaysia. Among the respondents, 50.2% were females. The majority of the respondents were 41–50 years old (27.8%), 27.8% belonged to the 51–60-year-old segment, 14.2% belonged to the 31–40-year-old segment, and 8.9% were more than 61 years old. The rest of the respondents in the study were below 30 years old, i.e., 4.7%. The majority (55.8%) of the respondents completed their primary school, and 30.2% had completed their secondary school level education. However, only 10.7% of respondents had an STPM level of education. The rest of the respondents never attended the school. The majority (42.7%) of the SMEs worked for 6–10 years, 32% of the SMEs worked for 11–15 years, 13.3% of SMEs worked for 16–20 years, and 11.6% of the SMEs worked for 1–5 years. Among the respondents, 59.1% had a service-oriented firm, 17.8% were engaged in retail, 11.6% worked in manufacturing, 7.3% were fishermen, 3.8% were livestock traders, and 0.4% were wholesalers. For the employment offered to the general public, 39.1% of the respondents had four employees, 21.6% of the respondents had three employees, 12.2% had five employees, 10.6% had more than six or more than six employees, two employees employed, 10.2% of the respondents, and 6% hired only one employee. Among the study respondents, 94% were married, and the remaining were single or divorced. The results are provided in Table 1.

**TABLE 1:** Profile of the respondent.

	*N*	%		*N*	%
***Gender***			***Firm established***		
Male	224	49.8	1–5 years	52	11.6
Female	226	50.2	6–10 years	192	42.7
Total	450	100.0	11–15 years	144	32.0
			16–20 years	60	13.3
*Age*			21 years and above	2	0.4
Up to 30 years old	21	4.7	Total	450	100.0
31–40 years old	64	14.2			
41–50 years old	200	44.4	***Types of firms***		
51–60 years old	125	27.8	Manufacturing	52	11.6
61 years old and above	40	8.9	Retailing	80	17.8
Total	450	100.0	Service	266	59.1
			Livestock	17	3.8
*Education*			Wholesaling	2	0.4
Never attended school	15	3.3	Fishing	33	7.3
Primary school	251	55.8	Total	450	100.0
Secondary school	136	30.2			
STPM/Diploma	48	10.7	***Number of employees***		
Total	450	100.0	None	2	0.4
			One	27	6.0
*Marital status*			Two	46	10.2
Married	423	94.0	Three	97	21.6
Single	22	4.9	Four	176	39.1
Divorced	1	0.2	Five	55	12.2
Widowed	4	0.9	Six and above	47	10.6
Total	450	100.0	Total	450	100.0

*Source: Author(s) own compilation.*

## Data Analysis and Results

### Reliability and Validity

The mean and standard deviation values of the sales orientation, customer orientation, competitive orientation, emotional orientation, business orientation, networking orientation, sustainability orientation, and SMEs sustainability are evaluated and reported in Table 2. The reliability and validity scores for the latent construct were assessed with the Cronbach Alpha (CA), Dillon-Goldstein rho (DG rho), and Composite Reliability (CR) and reported. Consequently, CA values are good above the 0.70 benchmarks (Chin, 2010), and the least CA score achieved value (0.613) by the business orientation. Next, the DG rho must be above 0.70 to depict sufficient reliability (Hair et al., 2019). The emotional orientation construct achieved the bottommost score (0.796). The CR also needs to be above 0.70 (Chin, 2010); the least score (0.767) realized by the business orientation construct for this study. The convergent validity was attained with the average variance extracted (AVE) value that needed to be higher than 0.50. all the AVE scores for latent constructs were above the 0.500 threshold (Hair et al., 2019). Finally, multicollinearity issues were estimated with the variance inflation factors (VIF). The VIF value of each factor is less than 3.3, suggesting that no significant collinearity/problem was present (Chin, 2010). Furthermore, the tolerance values were evaluated and reported to represent the non-significance of the collinearity issue in this study. The results are provided in Table 2.

**TABLE 2:** Reliability and validity.

Variables	Items	Mean	SD	CA	DG *rho*	CR	AVE	VIF	Tolerance
Sales orientation	7	3.34	1.008	0.933	0.964	0.943	0.705	1.858	0.519
Consumer orientation	4	4.00	0.607	0.832	0.969	0.877	0.648	2.462	0.442
Competitive orientation	4	3.97	0.715	0.825	0.891	0.886	0.668	2.145	0.440
Emotional orientation	4	4.03	0.718	0.727	0.796	0.819	0.534	1.773	0.528
Business orientation	3	3.73	0.739	0.613	0.950	0.767	0.537	1.578	0.630
Networking orientation	4	3.57	1.092	0.932	0.958	0.949	0.826	1.573	0.618
Sustainability orientation	3	4.22	0.580	0.765	0.781	0.863	0.677	1.000	0.678
Micro-enterprise sustainability	4	4.37	0.403	0.739	0.807	0.826	0.551	−	−

*SD, standard deviation; CA, Cronbach’s alpha; DG rho, Dillon-Goldstein’s rho; CR, composite reliability; AVE, average variance extracted; VIF, variance inflation factors.*

*Source: Author(s) own compilation.*

This study used cross-loading, Fornell-Larcker criterion, Hetro-trait, and Mono-trait (HTMT) ratio to appraise the discriminant validity (Hair et al., 2019). Lastly, discriminant validity for the study was further verified via a comparison between the loadings and cross-loadings for the constructs. Generally, loadings contribute an item to the latent variable to which it belongs (Hair et al., 2019), whereas cross-loading is the contribution of an item to other latent variables. Fornell-Larcker criterion was appraised by taking the square root of AVE of the construct, and the score must be greater than the corresponding correlation coefficient to establish the discriminant validity (Hair et al., 2019). This study constructs show suitable discriminant validity, as depicted in Table 3. Next, the study’s HTMT ratio was utilized to evaluate the discriminant validity (Henseler et al., 2015). All the HTMT ratios were less than the 0.900 bounds and professed that the latent construct achieved appropriate discriminant validity (Hair et al., 2019). The results are provided in Table 4.

**TABLE 3:** Loadings and cross-loadings.

Item code	SLO	CNO	CMO	EMO	BSO	NTO	SUO	MES
SLO – Item 1	**0.859**	0.471	0.463	0.465	0.380	0.198	0.413	0.210
SLO – Item 2	**0.871**	0.353	0.300	0.348	0.311	0.353	0.239	0.079
SLO – Item 3	**0.752**	0.164	0.110	0.233	0.087	0.434	0.094	−0.051
SLO – Item 4	**0.826**	0.287	0.171	0.331	0.219	0.369	0.190	−0.025
SLO – Item 5	**0.904**	0.311	0.292	0.340	0.319	0.353	0.367	0.128
SLO – Item 6	**0.801**	0.153	0.223	0.023	0.258	0.452	0.300	0.194
SLO – Item 7	**0.855**	0.329	0.365	0.327	0.362	0.311	0.366	0.187
CNO – Item 1	0.194	**0.550**	0.477	0.251	0.218	−0.077	0.077	0.136
CNO – Item 2	0.400	**0.899**	0.618	0.593	0.462	−0.160	0.567	0.385
CNO – Item 3	0.300	**0.906**	0.628	0.542	0.521	−0.207	0.455	0.369
CNO – Item 4	0.242	**0.812**	0.497	0.494	0.497	−0.199	0.257	0.269
CMO – Item 1	0.355	0.632	**0.911**	0.540	0.488	−0.243	0.526	0.417
CMO – Item 2	0.348	0.636	**0.911**	0.409	0.453	−0.191	0.450	0.460
CMO – Item 3	0.278	0.598	**0.852**	0.569	0.439	−0.230	0.417	0.334
CMO – Item 4	0.179	0.284	**0.536**	0.083	0.276	−0.042	0.232	0.321
EMO – Item 1	0.318	0.387	0.308	**0.803**	0.291	−0.048	0.503	0.176
EMO – Item 2	0.319	0.542	0.537	**0.685**	0.410	−0.202	0.194	0.231
EMO – Item 3	0.258	0.599	0.512	**0.799**	0.378	−0.243	0.387	0.311
EMO – Item 4	0.166	0.357	0.284	**0.621**	0.198	−0.072	0.212	0.160
BSOO – Item 1	0.314	0.486	0.435	0.355	**0.948**	−0.129	0.432	0.319
BSO – Item 2	0.245	0.456	0.520	0.331	**0.651**	−0.134	0.151	0.306
BSO – Item 3	0.293	0.293	0.245	0.330	**0.537**	0.021	0.117	0.075
NTO – Item 1	0.470	−0.026	−0.012	−0.032	0.100	**0.779**	−0.055	0.006
NTO – Item 2	0.372	−0.192	−0.236	−0.152	−0.107	**0.916**	−0.077	−0.130
NTO – Item 3	0.358	−0.197	−0.234	−0.162	−0.154	**0.962**	−0.120	−0.123
NTO – Item 4	0.330	−0.244	−0.266	−0.216	−0.173	**0.965**	−0.166	−0.168
SUO – Item 1	0.323	0.390	0.339	0.343	0.263	−0.071	**0.825**	0.359
SUO – Item 2	0.225	0.370	0.400	0.360	0.283	−0.162	**0.801**	0.377
SUO – Item 3	0.367	0.487	0.512	0.498	0.398	−0.094	**0.843**	0.460
MES – Item 1	0.116	0.356	0.415	0.260	0.308	−0.213	0.368	**0.837**
MES – Item 2	0.177	0.478	0.520	0.363	0.369	−0.250	0.495	**0.881**
MES – Item 3	0.084	0.110	0.160	0.071	0.122	0.114	0.321	**0.643**
MES – Item 4	0.064	0.073	0.163	0.060	0.105	0.144	0.172	**0.560**

*Bold indicates loading and others cross-loading.*

**TABLE 4:** Discriminant validities.

Fornell-Larcker criterion								
SLO	***0.840***							
CNO	0.375	***0.805***						
CMO	0.366	0.682	***0.817***					
EMO	0.365	0.621	0.529	***0.731***				
BSO	0.363	0.548	0.517	0.424	***0.733***			
NTO	0.392	−0.207	−0.234	−0.176	−0.128	***0.909***		
SUO	0.377	0.512	0.516	0.497	0.391	−0.131	***0.823***	
MES	0.161	0.397	0.467	0.295	0.338	−0.135	0.491	***0.742***
**Heterotrait-Monotrait ratio (HTMT)**							
SLO	−							
CNO	0.372	−						
CMO	0.364	0.804	−					
EMO	0.427	0.785	0.684	−				
BSO	0.467	0.752	0.742	0.701	−			
NTO	0.476	0.200	0.231	0.216	0.199	−		
SUO	0.389	0.511	0.617	0.570	0.439	0.139	−	
MES	0.170	0.391	0.550	0.367	0.424	0.271	0.595	−

*SLO, sales orientation; CNO, consumer orientation; CMO, competitive orientation; EMO, emotional orientation; BSO, business orientation; NTO, networking orientation; SUO, sustainability orientation; MES, micro-enterprise sustainability.*

*Source: Author(s) own compilation.*

*The Italic values in the matrix above are the item loadings, and others are cross-loadings.*

### Path Analysis

Attaining the acceptable construct level reliabilities and validities prompted us to estimate and report the model measurement level of evaluation. The adjusted *r*^2^ value for the five exogenous constructs (i.e., SLO, CNO, CMO, EMO, BSO, and NTO) on the sustainability orientation clarifies the 37.6% change in the firms’ level of sustainability orientation. The predictive relevance (*Q*^2^) value for the part of the model is 0.229, indicating a medium predictive relevance (Chin, 2010). The adjusted *r*^2^ value for the exogenous construct (i.e., sustainability orientation among the SMEs) on the SMEs’ sustainability elucidates the 24.1% change in the firms’ sustainability. The model fragment’s predictive relevance (*Q*^2^) value is 0.118, indicating medium predictive relevance (Chin, 2010).

Model standardized path values, *t*-values, and significance levels are shown in Table 5. The path coefficient between SLO and SUO (β = 0.202, *t* = 4.831, *p* < 0.001) indicates a significant and positive effect of SLO on the firms’ sustainability orientation, therefore support H1a. The path value for CNO and SUO (β = 0.129, *t* = 2.451, *p* = 0.007) illustrate the impact of CNO on the sustainability orientation, which is positive and significant; hence, it bids significant statistical support for H1b. The path between CMO and SUO (β = 0.203, *t* = 2.867, *p* = 0.002) shows the influence of CMO on the sustainability orientation as positive and significant; it offers provisions to admit the H1c.

**TABLE 5:** Path analysis.

Hypo		Beta	CI – Min	CI – Max	*T*	*P*	*r* ^2^	*f* ^2^	Q^2^	Decision
***Factors affecting Sustainability Orientation***										
H_1_	SLO → SUO	0.202	0.134	0.276	4.831	0.000		0.035		Accept
H_2_	CNO → SUO	0.129	0.047	0.219	2.451	0.007		0.011		Accept
H_3_	CMO → SUO	0.203	0.078	0.317	2.867	0.002	0.376	0.031	0.229	Accept
H_4_	EMO → SUO	0.199	0.113	0.275	4.073	0.000		0.036		Accept
H_5_	BSO → SUO	0.045	−0.054	0.159	0.702	0.242		0.002		Reject
H_6_	NTO → SUO	−0.095	−0.174	−0.018	1.991	0.023		0.009		Reject
***Factor affecting Micro-Enterprise Sustainability***										
H_7_	SUO → MES	0.491	0.491	0.046	10.748	0.000	0.241	0.317	0.118	Accept

*SLO, sales orientation; CNO, consumer orientation; CMO, competitive orientation; EMO, emotional orientation; BSO, business orientation; NTO, networking orientation; SUO, sustainability orientation; MES, micro-enterprise sustainability.*

*Source: Author(s) own compilation.*

The path value for EMO and SUO (β = 0.199, *t* = 4.073, *p* < 0.001) demonstrates the impact of EMO on the sustainability orientation as positive and significant; hereafter, it offers significant statistical support to accept the H1d. The path between BSO and SUO (β = 0.045, *t* = 0.702, *p* = 0.242) shows the influence of CMO on the sustainability orientation as positive but insignificant; it offers provisions not to accept the H1e. The path coefficient between NTO and SUO (β = −0.095, *t* = 1.991, *p* = 0.023) indicates a significant but negative effect of NTO on the firms’ sustainability orientation. The result forms no significant statistical support to accept the H1f. Lastly, the path coefficient between SUO and MES (β = 0.491, *t* = 10.748, *p* < 0.001) specifies a significant and positive effect of SUO on the firms’ sustainability, therefore support H2. The results are shown in Table 5.

### Mediation Analysis

The mediation effect of the SUO was tested with HM1 for the relationship between SLO and MES. The result (as presented in Table 6) reveals that the SUO significantly mediates the relationship between SLO and MES (β = 0.099, CI min = 0.063, CI max = 0.138, *p* < 0.001) and supports HM1. For HM2, the relationship between CNO and MES is mediated by SUO. The result displays that the SUO significantly mediates the relationship between CNO and MES (β = 0.063, CI min = 0.022, CI max = 0.114, *p* = 0.011); it offers sustenance to accept the HM2. For HM3, the relationship between CMO and MES is mediated by SUO. The result shows that SUO mediates the relationship between CMO and MES (β = 0.100, CI min = 0.038, CI max = 0.156, *p* = 0.002); it offers evidence to admit the HM3. For HM4, the relationship between EMO and MES is mediated by SUO. The result reveals that SUO mediates the relationship between EMO and MES (β = 0.098, CI min = 0.053, CI max = 0.142, *p* = 0.004); it affords provisions to admit HM4. For HM5, the relationship between BSO and MES is mediated by SUO. The result demonstrates that the SUO insignificantly mediates the relationship between BSO and MES (β = 0.022, CI min = −0.026, CI max = 0.079, *p* = 0.245); it offers sustenance to accept the HM5. For HM6, the relationship between NTO and MES is mediated by SUO. The result shows that SUO mediates the relationship between NTO and MES (β = −0.047, CI min = −0.088, CI max = −0.007, *p* = 0.027); it offers evidence not to acknowledge the HM6.

**TABLE 6:** Mediation analysis.

Mediating effect of sustainability orientation		Beta	CI – Min	CI – Max	*T*	*P*	Decision
H_8_	SLO → SUO → MES	0.099	0.063	0.138	4.384	0.000	Mediation
H_9_	CNO → SUO → MES	0.063	0.022	0.114	2.315	0.011	Mediation
H_10_	CMO → SUO → MES	0.100	0.038	0.156	2.832	0.002	Mediation
H_11_	EMO → SUO → MES	0.098	0.053	0.142	3.615	0.000	Mediation
H_12_	BSO → SUO → MES	0.022	−0.026	0.079	0.690	0.245	No mediation
H_13_	NTO → SUO → MES	−0.047	−0.088	−0.007	1.930	0.027	No mediation

*SLO, sales orientation; CNO, consumer orientation; CMO, competitive orientation; EMO, emotional orientation; BSO, business orientation; NTO, networking orientation; SUO, sustainability orientation; MES, micro-enterprise sustainability.*

*Source: Author(s) own compilation.*

## Discussion

This study empirically investigated the effect of six firm levels of strategic orientations (i.e., sales, consumer, competitive, emotional, business, and networking orientation) on sustainability orientation. The MEs’ sustainability orientation leads to ME sustainability among Malaysian MEs.

The first hypothesis confirmed that the sales orientation significantly instigates the sustainability orientation among the MEs in Kelantan, Malaysia. Our study outcome is consistent with the results posted by Panagopoulos et al. (2017) that the firm’s level of sales orientation harnesses the firm’s capacity to understand consumer interest and win the sales with the manipulative approaches that empower the firm to attain a sustainable mindset at the firm level. The following hypothesis gauged the effect of consumer orientation on the firm sustainability orientation for the MEs. The study finding suggests that the firms’ consumer orientation significantly influences the firm’s sustainability orientation level. Our study finding coincides with the outcome postulated by Kim (2017) that firms meet consumer expectations and positive business results that lead to the firms’ sustainability. The firm utilizes all available resources to cherish the consumer and empowers the firm to attain a sustainable competitive advantage (Thaiprayoon et al., 2019). Sustainability became a strategic asset by offering valuable service to the customer to gain the trust and loyalty of the consumers (Fernando et al., 2019).

The subsequent hypothesis assessed the firms’ competitive orientation promoted the firms’ sustainability. The study outcome revealed a significant positive consequence of competitive orientation on the sustainability orientation for the MEs. The current finding accords with the outcome posited by Salunke et al. (2019) that the firms’ capabilities and skills empower the firm to gain a better competitive market position and attain sustainability. Firms remain relevant and compete well in the highly competitive market (Na et al., 2019). The firms became adaptable and achieve high task orientation by incorporating flexibility (Klewitz, 2017). Next, our study evaluated and confirmed that emotion orientation’s vivacious and significant impact leads to achieving sustainability. The study outcome overlaps with the finding claimed by Hernandez-Perlines et al. (2019) that firms’ emotional orientation nurtures the firm’s sustainability. The emotional orientation helps achieve the creativity and business-level strategy formulation that enable the firms to perform well and attain sustainability as a business has a higher emotional attachment by the firm management and staff (Berrone et al., 2012).

The following hypothesis measured the firms’ business orientation and encouraged the firm-level of sustainability. This study as a consequence exposed an insignificant positive consequence of business orientation on the sustainability orientation. Our study’s result does not agree with Fernando et al.’s (2021) that the business orientation necessary for improving the business operation leads to better business performance and sustainability. Business orientation was weak among the Malaysian MEs and did not appropriately facilitate the firms performing and attaining sustainability. The business orientation requires taking care of all aspects of business-like technology, process, environment, and society (Chang, 2016), where the Malaysian firms are not fully grabbing the essence of the business orientation. The narrow point of view of business orientation only can promote the marketing or meet the customer orientation (Behnam and Cagliano, 2019). Subsequently, our study appraised and reported a negative but significant effect of networking orientation on the firms’ sustainability orientation. The study outcome contradicts the postulation made by Su et al. (2015) that the networking orientation helps the firms perform better and enable them to gain sustainability. The networking orientation empowers the firm to choose and work with the right business partners and achieve superior performance (Jones et al., 2017). However, Malaysian MEs are not fully aware of networking benefits that bring superior production and service delivery. Superior performance is only achieved by the positive collective interaction of business partners forming the network to perform and sustain in a highly competitive marketplace (Klewitz, 2017).

Finally, our study revealed a significant positive effect of sustainability orientation on the MEs’ sustainability. The finding concurs with Adedeji et al. (2019b), thus confirming a significant and positive influence of sustainability orientation on the MEs’ sustainability (i.e., economic, social, and environmental performance). Based on the finding, we conclude that a positive orientation (of not harming) toward the environment and society facilitates achieving MEs sustainability.

For the mediating analysis, the finding of the analysis revealed a significant meditational effect of sustainability orientation between the sales orientation and MEs sustainability and accepted the HM1. The sales mindset favors the firm to work closely with designing acceptable products and services that instigate the sustainable mindset that leads to the sustainable performance of the firm (Panagopoulos et al., 2017). The following mediating hypothesis was proposed to evaluate the mediating effect of sustainability orientation between consumer orientation and MEs sustainability. The result confirms the significant mediating effect of sustainability orientation for the said relationship. Our finding coincides with the result posted by Khan and Khan (2019) that the consumer orientation empowers the firm to meet the customer expectation and achieve sustainability productively. The firm-level sustainability promotes the firm’s sustainability. Subsequent mediating hypothesis projected to appraise the mediating effect of sustainability orientation between the relationship of competitive orientation and MEs sustainability. The outcome settled the substantial mediating effect of sustainability orientation for the supposed association. The study outcome matches with the finding forwarded by Kim (2017) that the competitive mindset builds the sustainability orientation for the firm and leads to the firm’s sustainability. Following the mediating analysis, the result confirms the noteworthy mediating effect of sustainability orientation on the alleged relationship. Our finding agrees with the result posted by Hernandez-Perlines et al. (2019) that the emotional orientation allows the firm to productively engage with the firm’s activities and bring positive business results. Next, the mediating hypothesis was projected to judge the mediating effect of sustainability orientation between the relationship of business orientation and MEs sustainability. The result confirms the inconsequential mediating effect of sustainability orientation for the hypothetical relationship. Our finding challenges the result posted by Adiguzel (2019) that the business mindset permits the firm to engage in business activities productively. The sustainability orientation harnesses stable business returns that lead to sustainable business performance (Khizar et al., 2022). Lastly, the mediating hypothesis is projected to judge the mediating effect of sustainability orientation between network orientation and MEs sustainability. The result confirms the insignificant mediating effect of sustainability orientation on the supposed relationship. Our finding disagrees with the outcome posted by Klewitz (2017) that the network mentality permits the firm to participate in business operations with the stakeholder productively, leading to superior business performance.

## Implications, Limitations, and Future Direction and Conclusion

### Theoretical Implications

For the theoretical dominion, this study contributes explicitly to the RBV by establishing the identified aspects of strategic orientation as unique and valuable efforts or resources that could be explored to operationalize sustainability-oriented business production processes that lead to superior social, economic, and environmental firm performance among the MEs. The study results add to the empirical evidence that the personal inclination depicted as MEs orientation and business attitude take the business MEs. In the emerging economy, the sales, customer, competitive, and emotional orientations nurture the sustainable orientation, harnessing the sustainable performance for the MEs. The business and networking orientations were weak among the MEs working in emerging economies and did not effectively connect with the MEs’ level of sustainability orientation and sustainable performance. Finally, the RBV framework is widely used by MEs or entrepreneurial firms to investigate and suggest improvements to the working and performance of the MEs.

### Practice Implications

This study’s findings can support policymakers and socio-developmental entities to help and nurture the MEs’ capacities to develop and formulate firm-level policies and programs that can strengthen sales, consumer, competitive, and emotional orientation among MEs in Malaysia. This study offers significant practice contributions in that MEs are mostly entrepreneurial enterprises and are actively engaged in a sustainable mindset. Nevertheless, the capacity building and effective execution of MEs’ sustainability initiatives can promote the MEs’ performance in emerging economies. Finally, for owners and managers of MEs, the current work also highlights the importance of taking firm-level strategic orientation to empower the firm’s sustainable performance. Therefore, it is recommended that MEs advance and thus leverage MEs’ strategic orientation dimensions instead of depending on government and non-government support for cultivating MEs’ sustainability, which could lead to the better socioeconomic performance of MEs.

### Limitations and Future Directions

We acknowledge using a dataset acquired from a single state in Malaysia focusing only on MEs (one firm size) for the study limitations. Future researchers could consider a more inclusive and geographically diverse dataset to overcome the limitation. Second, the strategic orientation dimensions could be industry-specific; the study (using respondents from several industries) portrays a general list of orientations that could be exploited to achieve sustainability. Future researchers should focus on specific industries separately to reveal orientation dimensions relevant to the respective industry. Lastly, using the same model in different geographic locations can help establish the empirical support for the influence of strategic orientation empowering the sustainable performance of MEs.

## Conclusion

This study aims to evaluate the influence of the strategic orientations (sales, consumer, competitive, emotional, business, and networking) on the sustainability orientation and promote the MEs’ sustainable performance. The study findings revealed significant positive effects of sales orientation, consumer orientation, competitive orientation, and emotional orientation on the MEs’ sustainability orientation in Kelantan, Malaysia. The mediational analysis confirms the mediating role of sustainability orientation in the relationship between sales, consumer, competitive, and emotional orientation on MEs’ sustainability. The study results suggest that more effort must be undertaken by MEs’ top management to work on improving the business and networking efforts. The MEs need to evaluate and change their business and networking mindsets. The attainment of an appropriate orientation can empower the MEs to achieve a sustainable mindset and sustainable performance. This is a common misunderstanding.

## Data Availability Statement

The original contributions presented in the study are included in the article/Supplementary Material, further inquiries can be directed to the corresponding author.

## Ethics Statement

Ethical review and approval was not required for the study on human participants in accordance with the local legislation and institutional requirements. The patients/participants provided their written informed consent to participate in this study. Written informed consent was obtained from the individual(s), and minor(s)’ legal guardian/next of kin, for the publication of any potentially identifiable images or data included in this article.

## Author Contributions

WW, NH, SF, and AA: conceptualization, methodology, questionnaire design, and data collection. NH, AA, and AS: data analysis, initial draft, and review. NH, AA, and NZ: formal analysis, review, and updates. All authors contributed to the article and approved the submitted version.

## Conflict of Interest

The authors declare that the research was conducted in the absence of any commercial or financial relationships that could be construed as a potential conflict of interest.

## Publisher’s Note

All claims expressed in this article are solely those of the authors and do not necessarily represent those of their affiliated organizations, or those of the publisher, the editors and the reviewers. Any product that may be evaluated in this article, or claim that may be made by its manufacturer, is not guaranteed or endorsed by the publisher.

## Footnotes

1
https://webpower.psychstat.org/wiki/tools/index

## Appendix

**APPENDIX 1:** Survey instrument.

Questions	
SLO – Item 1	Begin the sales talk for a product before exploring a customer’s need.
SLO – Item 2	Try to sell a customer all I can convince him/her to buy even if I think it is more than a wise customer would buy
SLO – Item 3	Try to sell as much as I can rather than satisfy a customer
SLO – Item 4	Stay alert for weaknesses in a customer’s personality so I can use them to put pressure on his/her to buy
SLO – Item 5	Will apply pressure to get a customer to buy a product, even if I am not sure that it is suitable for him/her
SLO – Item 6	Paint too rosy a picture of my product to make them sound as good as possible
SLO – Item 7	Spend more time trying to persuade a customer to buy rather than I do try to discover his/her needs
CNO– Item 1	Consciously seeks to identify and acquire new customers individually
CNO– Item 2	Analyze past customer transactions at the individual level to predict future transactions from that customer
CNO– Item 3	Encourage the customer to share opinions of its product or services
CNO– Item 4	Predict what each individual customer will contribute to its profit in the future
CMO – Item 1	Competitions in our industry is quite intense
CMO – Item 2	There are many sales-promotions campaigns in our industry
CMO – Item 3	Anything one competitor can offer others can match readily
CMO – Item 4	One hears of a new competitive move almost every day
EMO– Item 1	When I am faced with business-related obstacles, I remember times I faced a similar obstacle and overcame them
EMO– Item 2	I like to share my emotions with my suppliers and customers
EMO– Item 3	I am aware of the non-verbal messages I send to suppliers and customers
EMO– Item 4	I recognize the emotions that people are experiencing by looking at their facial expressions
BSO– Item 1	Have launched many new products/services on the market during the last 5 years
BSO– Item 2	Usually, beat our competitors in developing innovative actions
BSO– Item 3	When uncertainty is high, I adopt a brave and aggressive attitude to exploit possible opportunities
NTO – Item 1	I have a high number of business network partner
NTO – Item 2	My network is very diverse
NTO – Item 3	My network partner frequently provides me with new information
NTO – Item 4	I receive extensive support from my network partners
SUO – Item 1	Have knowledge about the new product and services offered by competitors
SUO – Item 2	Have knowledge about the issues concerning the business environment
SUO – Item 3	Focused on the overall development of the local community
MES – Item 1	Employee satisfaction
MES – Item 2	Retention of employees
MES – Item 3	Social reputation
MES – Item 4	Investment in society
